# Novel mutation in TENM3 gene in an Iranian patient with colobomatous microphthalmia

**DOI:** 10.1002/ccr3.5532

**Published:** 2022-03-08

**Authors:** Sepideh Gholami Yarahmadi, Fatemeh Sarlaki, Saeid Morovvati

**Affiliations:** ^1^ School of Advanced Sciences and Technology Islamic Azad University‐Tehran Medical Sciences Tehran Iran; ^2^ 556492 Shahid Beheshti University of Medical Sciences Tehran Iran

**Keywords:** coloboma, gene, microphthalmia, mutation, novel, TENM3

## Abstract

This investigation revealed a homozygous c.5069‐1G>C variation in TENM3 gene although has not been reported for its pathogenicity and can be considered as a novel mutation. The present finding can be used for genetic diagnosis and detection of carriers in the family and other patients with similar disease manifestations.

## Graphical abstract

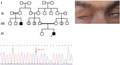

## 1 INTRODUCTION

Microphthalmia, anophthalmia, and coloboma (MAC) display a range of MAC ocular malformations.^1^ The conditions of MAC are mostly related to further ocular and nonocular anomalies, demonstrating the gene association accountable for several processes of development. It is reported that almost 33% of reported cases affected with MAC are syndromic and have abnormalities such as the craniofacial, renal, genital, cardiac, brain, and skeletal.^2^

Anophthalmia (AO), MIM 206900, and microphthalmia (MO), MIM 309700, are the worst congenital deformities of the eye in terms of severity, with a prevalence of around 1 in 30,000 and 1 in 7000 births, in turn.^3^, ^4^, ^5^ AO refers to the complete absence of the optic tissue structure,^6^, ^7^ or the structures of visible ocular with remnants that can be detected histologically.^8^ MO is defined as a decrease in the ocular globe size (total axial length of <19 mm in 1‐year‐old children and <21 mm in adults).^6^, ^9^, ^10^

These defects can be syndromic or isolated^11^, ^12^ and may occur unilateral or bilateral^6^ with abnormalities occurring in the vitreous (persistent fetal vasculature), lens (congenital cataract), anterior segment (sclerocornea or Peters anomaly, microcornea, iris coloboma), and/or posterior segment (optic coloboma).^13^, ^14^, ^15^, ^16^, ^17^

Microphthalmia can be categorized into simple MO and complex MO based on the presence of other ocular malformations or systemic diseases. The simple MO is defined as an eye reduced in size but with normal shape, except for the short axial length.^10^ In comparison, the complex MO occurs along with other eye deformities, such as chorioretinal coloboma, iris coloboma, retinal coloboma, and persistent fetal vasculature.^12^, ^14^, ^18^, ^19^

Based on epidemiological studies, AO and MO have both heritable and environmental causes, with genetic defects being the majority of common causes.^12^, ^14^, ^19^, ^20^ Beyond 30 genes are associated with the nonsyndromic AO and MO pathogenesis, the main causative of which are *RAX* (MIM 601881), *OTX2* (MIM 600037), *PAX6* (MIM 607108), *FOXE3* (MIM 6011094),^14^, ^20^ and *SOX2* (MIM 184429).^21^

Based on the reports, several chromosomal abnormalities such as trisomy 13, mosaic trisomy 9, del7p15.1‐p21.1, del14q22.1q23.2, delXp22.3, del16p11.2, del16q11.2q12.2, dup10q24.31, and dup15q11.2q13.1, and also some point mutations are involved in MO. The rearrangement of chromosomes has been identified mainly related to syndromic MO, while single nucleotide variants could be detected in both nonsyndromic and syndromic forms.^20^, ^22^ As several genes are involved in most cases of chromosomal rearrangements, the resulting disorder is usually syndromic, while disorders caused by point mutations can be syndromic or nonsyndromic, depending on the type of mutations and involved genes. Due to the incidence of de novo mutations, incomplete penetrance, mosaicism, and sporadic occurrence, genetic counseling is not easy.^3^

In this study, we investigated the genetic basis of microphthalmia in an affected Iranian proband and reviewed the reported spectrum of the TENM3 gene mutations involved in this disorder.

## 2 MATERIAL AND METHODS

**FIGURE 1:**
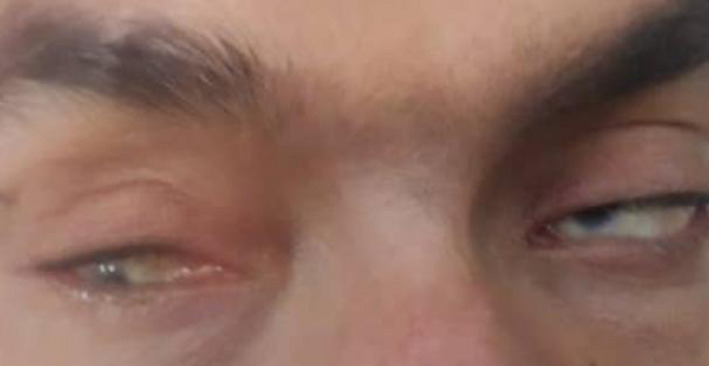
Photograph of patient's eyes

**FIGURE 2:**
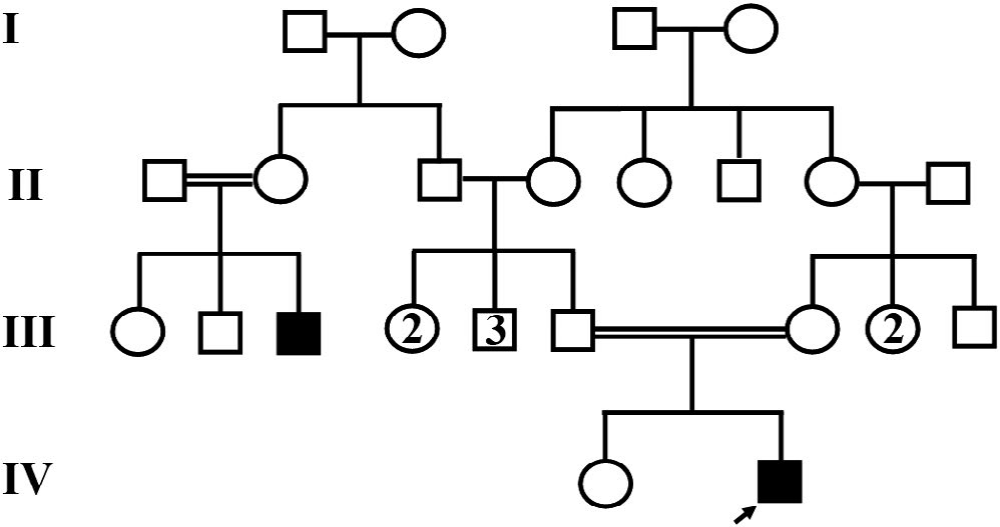
Family pedigree of the patient

A 32‐year‐old symptomatic male with mild intellectual disability, bilateral decrease in the ocular globe size, coloboma, glaucoma, and cataract, living in Sari city of Iran diagnosed as having bilateral colobomatous microphthalmia based on his clinical and paraclinical features. (Figure 1). His parents were first cousins, and there was a positive family history in his pedigree. First cousin of our patient's father (case III‐3) is also affected by Mo and Coloboma, without intellectual disability (The patient did not consent to the genetic test). After genetic counseling and drawing the familial pedigree (Figure 2), the proband gave his informed consent before the inclusion in this experiment. DNA extraction was done from whole blood using standard extraction methods. Human whole‐exome enrichment was performed using Twist Human Core Exome Kit, and the library was sequenced on Illumina platform with a raw coverage of 260X and mean on‐target coverage of 105X, performed by CeGaT GmbH, Germany. Only data related to the 35 genes of interest were extracted for further analysis (Name of these genes and their inheritance patterns are mentioned in Table 1 based on OMIM databases). Our panel of 35 genes is based on the genes listed in the OMIM Database for this disease, including genes that cause the isolated disease and genes that cause the syndromic type. For each disease, a panel of genes is introduced in the OMIM database, and in the study of that disease, all those genes are examined, whether they are the cause of syndromic or cause of isolated type. On the other hand, because it was possible that the patient's intellectual disability was not associated with microphthalmia, we examined both isolated and syndromic‐type causative genes. Nearly all exons and flanking 10bp in these genes were detected and analyzed. The NGS method's analytical sensitivity and specificity used in this assay to detect single point mutations and small indels (within 20 bp) are assumed to be >95%.

**TABLE 1:** Checked genes related to microphthalmia

Number	Official symbol	Inheritance	MIM number	Number	Official symbol	Inheritance	MIM number	Number	Official symbol	Inheritance	MIM number
1	*ABCB6*	AD	605452	13	*HCCS*	XLD	300056	25	*RAX*	AR	601881
2	*ALDH1A3*	AR	600463	14	*HESX1*	AD, AR	601802	26	*SHH*	AD	600725
3	*BCOR*	XLD	300485	15	*IKBKG*	XLD, XLR	300248	27	*SIX6*	AR	606326
4	*BEST1*	AD	607854	16	*MFRP*	AR	606227	28	*SMOC1*	AR	608488
5	*B3GALNT2*	AR	610194	17	*MKS1*	AR	609883	29	*SOX2*	AD	184429
6	*BMP4*	AD	112262	18	*NDP*	XLD, XLR	300658	30	*STRA6*	AR	610745
7	*CHD7*	AD	608892	19	*OTX2*	AD	600037	31	*TENM3*	AR	610083
8	*COX7B*	XLD	300885	20	*PAX2*	AD	167409	32	*TMEM67*	AR	609884
9	*ERCC6*	AR	609413	21	*PAX6*	AD	607108	33	*VAX1*	AR	604294
10	*ERCC8*	AR	609412	22	*PITX3*	AD, AR	602669	34	*VSX2*	?	142993
12	*GDF3*	AD	606522	23	*POMT1*	AR	607423	35	*NAA10*	XL	300013
11	*GDF6*	AD	601147	24	*PRSS56*	AR	613858				

Abbreviations: AD, autosomal dominant; AR, autosomal recessive; XLD, X‐linked dominant.

## 3 RESULT

The proband described in this study had clinical manifestations such as mild intellectual disability, bilateral decrease in the ocular globe size, and coloboma, which conform to the diagnosis of nonsyndromic bilateral colobomatous microphthalmia. Both parent's detailed ocular examination was normal. The patient's parents were normal based on eye examinations performed by a specialist physician.

**FIGURE 3:**
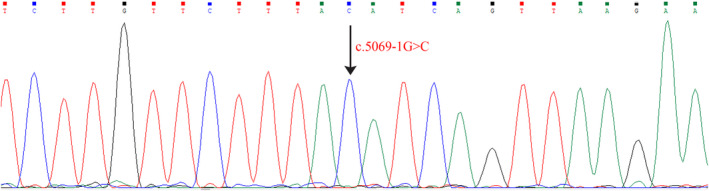
Chromatogram is showing the homozygous mutation c.5069‐1G>C in the *TENM3* gene in the patient

Sanger validation of the *TENM3* gene endorsed the fact that the proband had a homozygous c.5069‐1G>C variation (Figure 3). The detected homozygous canonical splice site variant in the *TENM3* gene has not been reported up to now for its pathogenicity. However, based on various silico computational analyses mentioned in the Varsome database for pathogenicity scores such as BayesDel addAF, BayesDel noAF, DANN, EIGEN, EIGEN PC, FATHMM‐MKL, and Mutation Taster, the variant has a deleterious effect on the gene or gene product(s). Founded on the American College of Medical Genetics and Genomics (ACMG) guideline, this variant can be categorized as pathogenic (PVS1: Very Strong, PM2: Moderate, PP3: Supporting).

## 4 DISCUSSION

Congenital malformation of the eye is one of the main reasons for blindness and ocular morbidity in childhood. Considering almost 4000 genetic disorders and syndromes, which have an effect on humans, at least 33% affects the eye.^23^

The *TENM3* gene encodes the Teneurin transmembrane protein 3 in humans, which has been investigated for its role in the development of the eye, adhesion of homophilic cells, and axon guidance.^24^, ^25^ This protein consists of 2699 amino acids (NP_001073946.1). The family of Teneurin includes 4 distinct types of transmembrane dimeric proteins (*TENM1*‐*4*).^26^

It has been shown that the *TENM3* gene expresses in the nervous system and a restricted set of mesoderm‐derived tissues. It has been suggested that the *TENM* gene plays a vertebrate orthologue conserved role in ocular development as it was detected to be mainly enriched in the optic stalk.^24^ Due to the strong brain teneurins expression in neuronal subpopulations and the positional mapping, there could be a connection to intellectual disability, especially during development.^27^

To the best of our knowledge, seven mutations have been reported in the *TENM3* gene in 6 unrelated families, 6 of which are ascribed eye anomalies. Our report would be the 7^th^ MO and coloboma causative mutation in this gene.^2^, ^26^, ^28^, ^29^, ^30^, ^31^ You can see the information of these seven mutations in Table 2.

**TABLE 2:** Characteristics of reported mutations involved in microphthalmia

Clinical characteristics	^28^		^2^	^29^	^26^		^31^	This study
Mutation	Homozygous c.2083dup; p. Thr695Asnfs*5		Homozygous c.2968‐2A>T; p. Val990Cysfs*13	Compound heterozygous c.7687C>T; p. Arg2563Trp and c.4046C>G; p. Ala1349Gly	Homozygous c.1857T>A; p. Cys619*		Homozygous c.1558C>T; p.(Arg520*)	Homozygous c.5069‐1G>C p.1690D>Gfs*2
Type of mutation	Frameshift		Splice	Missense	Nonsense		Nonsense	frameshift
Exon/intron containing mutation	Exon 12		Intron 16	Exon 22 and exon 28	E11		E9	Intron 23
Consanguinity	Yes		Yes rep04	No INTELL1	NO 16		Yes 017	Yes
Origin	Saudi Arabia		France	India	India		Pakistan	Iran
Gender	Male	Female	Male	Male	Female	Female	Not given	Male
Age	11	9	9	6	5 years and 6 months	3 years and 4 months	Not given	32
Motor development	Normal		Delayed	Delayed	Delayed	Delayed	Not given	Normal
Cognition	Normal		Delayed	Delayed	Delayed	Normal	Not given	Delayed
Ptosis	No		No	No	Unilateral (left)	Bilateral partial ptosis	Not given	yes
Microphthalmia	Yes		Yes	Yes (right eye)	No	No	Yes	Yes
Micro cornea	Yes		Yes	Bilateral sclerocornea	Yes	Yes	Not given	?
Corneal shape	Oval	Not given	Not given	Not given	Vertically oval	Vertically oval	Not given	?
Iris coloboma	Inferior	Inferior	Inferior	Not given	Inferonasal	Inferonasal	bilateral iris and chorioretinal colobomas	?
Shape of disk	Anomalous	Not given	Not given	Not given	Normal	Normal	Not given	?
Disk coloboma	Yes	Yes	Yes	Not given	Inferonasal bilateral involving fovea	Inferonasal bilateral involving fovea	?	?
Visual acuity	20/50(R) Hand movement (L)	20/200(R) 20/300(L)	Hand movement both eyes	Not given	6/36 both eyes	6/36 both eyes	Not given	?

The first mutation of the *TENM3* gene was reported in two siblings of a consanguineous family. These brothers were both suffering from isolated bilateral microphthalmia, microcornea, and retinal and iris coloboma. The homozygous c.2083dup variant was detected in them, while their parents were unaffected carriers.^28^

A homozygous splice mutation (c.2968‐2A>T) in the *TENM3* gene was detected in a son of 9 from a consanguineous family. The proband was affected by bilateral colobomatous microphthalmia and developmental delay.^2^

Two novel compound heterozygous variations (c.4046C>G and c.7687C>T) in the *TENM3* gene was found in a boy of 6, with eye anomalies and intellectual disability.^29^

Another novel mutation (c.1857T>A) in the homozygous state in the *TENM3* gene has been reported in two sisters from nonconsanguineous parents. These siblings did not have microphthalmia, but they had ptosis, developmental delay, and iris coloboma.^26^

Feldman et al^30^. found a homozygous c.7994A>C variant in the *TENM3* gene in three affected patients of a 4‐generation family who were suffering from developmental dislocation of the hip.

In addition, Islam et al^31^. identified c.1558C>T (a pathogenic homozygous variant) in the *TENM3* gene in a patient who was suffering from cataracts, bilateral iris, and chorioretinal colobomas microphthalmia.

Therefore, it seems that the *TENM3* gene is vital in the eye development process, and pathogenic variations of this gene could bring about MAC ocular malformations spectrum and intellectual disability. The detected mutation in our case, c.5069‐1G>C, has not been reported before and can be considered as a novel mutation. The present finding can be used for genetic diagnosis and detection of carriers in the family and other patients with similar disease manifestations.

## CONFLICT OF INTEREST

No conflict of interest is hereby declared by any of the contributing authors.

## AUTHOR CONTRIBUTIONS

Sepideh Gholami Yarahmadi: performed genetic laboratory tests, data analysis, sampling, original draft. Fatemeh sarlaki: involved in investigation and resource. Saeid Morovvati: involved in conceptualization, writing—review and editing, supervision, and formal analysis.

## ETHICAL APPROVAL

This study was approved by the ethical committees of Rasad Pathobiology and Genetic Laboratory, Tehran, Iran. Written informed consent to participate for genetic studies was obtained from the patients of this study.

## CONSENT

The patient has provided us with his written consent for publishing this study, and the study was conducted according to the Helsinki Declaration principles.

## Data Availability

The data that support the findings will be available in www.Figshare.com https://figshare.com/s/c6d549b63c9d62f3d34b following an embargo from the date of publication to allow for commercialization of research findings.
